# Gender Oncology: Rethinking Cancer Through a Sex- and Gender-Based Lens

**DOI:** 10.3390/jcm15145435

**Published:** 2026-07-11

**Authors:** Rossana Berardi, Carolina Liguori, Francesca Rossi

**Affiliations:** Department of Oncology, Università Politecnica delle Marche, AOU Marche, 60126 Ancona, Italy; liguoricarolina95@gmail.com (C.L.); rossifrancesca561@gmail.com (F.R.)

**Keywords:** gender oncology, sex differences, cancer, epidemiology, outcome, toxicity, anticancer therapies

## Abstract

**Background**: Gender medicine is an emerging approach that recognizes how biological sex, gender identity, and sociocultural roles influence health and disease. These factors can significantly affect prevention, screening, diagnosis, and treatment outcomes. In oncology, accumulating evidence highlights sex-based differences in cancer incidence, therapeutic response, and treatment-related adverse events. A comprehensive gender-based approach should also address the specific needs of sexual and gender minority (SGM) populations. Based on the existing literature and the authors’ expertise, this perspective paper provides an overview of this topic, emphasizing its importance, discussing current challenges, and proposing potential future directions for research and clinical practice. The literature discussed in this article was selected through a narrative, rather than a systematic, approach and is intended to provide the scientific context supporting the authors’ perspective and interpretation of the current evidence. **Methods**: To investigate gender differences in oncology regarding epidemiological data, we queried the AIRTUM and GLOBOCAN databases. Furthermore, we conducted a bibliography search of scientific papers by querying PubMed/MEDLINE to explore gender differences, in particular regarding treatments. The database was searched by looking for papers published between 1 January 2022 and 31 December 2025. **Results**: Cancers affecting both sexes show higher incidence and mortality rates in men, a trend observed worldwide and confirmed by national data. However, important sex-related differences also emerge in treatment response. Women appear to derive a smaller survival benefit than men from immunotherapy alone but may experience greater benefit when immunotherapy is combined with chemotherapy. These differences may be explained by distinct molecular mechanisms underlying antitumor immune responses in males and females. Furthermore, women experience higher rates of treatment-related toxicities, likely due to sex-specific differences in pharmacokinetics and pharmacodynamics. **Conclusions**: Integrating sex and gender perspectives into oncology is essential for advancing personalized medicine and improving patient care. Understanding biological differences can help optimize treatment selection and dosing strategies, thereby enhancing efficacy while minimizing adverse effects. At the same time, addressing gender-related factors may improve psychosocial support, patient well-being, and treatment adherence. Achieving these goals requires coordinated efforts, including sex-disaggregated research, gender-sensitive clinical protocols, and healthcare policies aimed at reducing gender disparities in cancer care.

## 1. Introduction

Over the past decades, oncology has entered the era of precision medicine. Advances in genomic profiling and the development of molecularly targeted therapies have transformed cancer care, enabling increasingly personalized treatment strategies. However, the influence of sex and gender has not yet been systematically integrated into oncological research and clinical practice, despite long-standing recognition of their role as fundamental determinants of health and disease [1]. Biological sex influences every aspect of tumor biology, including incidence, pathogenesis, and clinical manifestations. It also affects treatment efficacy and adverse events. At the cellular level, genetic, epigenetic, hormonal, and environmental determinants contribute to the distinct biological behavior of male and female cells [2,3]. Despite this knowledge, preclinical and clinical research have long relied on male models—using male animals in experimental studies due to concerns about hormonal variability and recruiting predominantly male participants in clinical trials [4,5]. Minor inclusion of women in trials leads to therapeutic strategies that may not fully account for female physiology. Although in recent years the concept of sex- and gender-inclusive research has emerged, its practical implementation remains limited [6]. Gender differences are not limited to biology but extend into social and cultural contexts. These also influence cancer risk and diagnosis, access to healthcare, and adherence to therapy [7]. Together, sex and gender present a challenge to the current modern oncology paradigm, which aims to overcome the ‘one-size-fits-all’ approach. This perspective paper, supported by a narrative review of the existing literature, explores the biological and sociocultural foundations of sex- and gender-based differences in oncology. The literature was selected to provide the scientific context for the authors’ perspective rather than through a systematic review methodology. Based on this evidence and the authors’ expertise, the paper argues that precision medicine must reflect the full spectrum of human diversity. Improving gender awareness in cancer care is a matter not only of scientific rigor but also of fairness and dignity for all people living with cancer.

## 2. Materials and Methods

To investigate sex- and gender-related differences in oncology from an epidemiological perspective, we analyzed data from the AIRTUM and GLOBOCAN databases. Furthermore, we conducted a literature search in PubMed/MEDLINE to explore gender differences, in particular in cancer treatments with attention to outcomes and therapy-related toxicities. The search strategy involved the use of several queries (keywords/MESH terms). The database search was restricted to articles published between 1 January 2022 and 31 December 2025, reflecting the recent growth of evidence demonstrating sex-related differences in cancer incidence, treatment response, and therapy-associated adverse events. The following inclusion criteria were applied: studies published between 1 January 2022 and 31 December 2025; articles written in English or Italian; studies involving healthy adults or adults with cancer; and studies relevant to the Italian healthcare context. Exclusion criteria were publications in languages other than English or Italian; studies published outside the selected time frame; studies involving pediatric populations; studies focused on non-oncological or hematological diseases; and studies not relevant to the field of gender oncology.

## 3. Differences in Epidemiology

According to 2024 estimates, cancer prevalence in Italy reached approximately 3.7 million individuals (6.2% of the population). Of these, 56% were women (6.8% of the total female population) and 44% were men (5.6% of the male population). Among men, prostate cancer was the most prevalent malignancy, followed by colorectal and bladder cancers. In women, breast cancer accounted for the highest prevalence, representing nearly half (45%) of all female cancer survivors. Other highly prevalent cancers in women included those of the colorectum, thyroid, and endometrium. According to estimates in 2024 in Italy, there were 390,000 new cases of cancer (excluding skin tumors other than melanoma), specifically 214,000 new cases in men and 175,000 new cases in women. Among men, the most frequently diagnosed cancers were prostate (40,190 new cases), lung (31,900), colorectal (27,500), bladder (25,230), and kidney cancers (8890). Among women, breast cancer was by far the most common (53,060 new cases), followed by colorectal (21,230), lung (12,940), endometrial (8650), and thyroid cancers (8320) [8].

In men, in the period 2007–2019, for all cancer sites combined, there were an estimated 206,238 fewer deaths than expected, equivalent to a 14.4% decrease in cancer deaths over the entire period. In women, there were an estimated 62,233 fewer deaths than expected, equivalent to a 6.1% decrease in cancer deaths over the entire period [9].

Worldwide, the incidence rate for all combined cancers was 14% higher in men (212.6 per 100,000 py) than in women (186.3 per 100,000 py) in 2022, with some regional oscillations.

Worldwide, the incidence rate for all combined cancers was 14% higher in men (212.6 per 100,000 person-years) than in women (186.3 per 100,000 person-years) in 2022, with some regional variations. The gender gap in overall cancer mortality worldwide was approximately twice that observed for incidence, with mortality rates 43% higher in men than in women (109.8 and 76.9 per 100,000, respectively) [10]. This discrepancy suggests that factors beyond differences in cancer occurrence contribute to the observed survival disadvantage in men. In part, it reflects the different distribution of cancer types between sexes, with men more frequently affected by highly lethal malignancies such as lung, liver, and bladder cancers. In addition, men are often diagnosed at more advanced stages, partly due to lower participation in preventive and screening programs and delays in seeking medical attention. Differences in exposure to environmental and behavioral risk factors, as well as inequalities in access to timely diagnosis and high-quality treatment, may further contribute to the wider mortality gap observed between men and women.

Several studies have shown significant differences between men and women in the stage at which various cancers are diagnosed, with women generally presenting at earlier stages than men [11,12,13]. Since the stage at diagnosis is a major determinant of cancer-related mortality, these differences are likely to contribute to the lower mortality observed in women.

The underlying causes are thought to involve both biological (sex-related) and sociocultural (gender-related) factors. The following paragraphs first discuss gender-related determinants influencing cancer diagnosis and healthcare utilization, followed by biological mechanisms that may contribute to sex-related differences in cancer susceptibility and progression.

In some cancers, differences in perceived risk and referral practices may influence the timing of diagnosis. Women are also more likely to perform self-examinations, which may facilitate earlier detection of visible signs of cancer such as melanoma lesions or palpable lumps [14]. Gender differences in health-seeking behavior also play a role. Women are more likely to participate in screening programs [15] and to seek medical advice promptly in response to symptoms [16]. In contrast, delays in care among men have been attributed to gender norms emphasizing “machismo” and the perception that medical visits are inconvenient [17,18]. Conversely, in many developing countries, socioeconomic and cultural disempowerment can limit women’s access to healthcare [19,20,21].

However, differences in health-seeking behavior, lifestyle factors such as diet, smoking, alcohol consumption, and environmental exposures do not appear to fully account for the observed sex disparities in cancer epidemiology. This has prompted investigations into biological mechanisms that may contribute to these differences, although the relative importance of individual mechanisms and their contribution to population-level outcomes remains incompletely understood.

Men are generally more exposed to environmental carcinogens such as tobacco smoke and occupational pollutants, whereas women exhibit distinct hormonal and genetic characteristics that may influence cancer susceptibility and progression. Several biological mechanisms have been proposed to explain these differences. For example, the presence of two X chromosomes in females may provide protection through the expression of tumor suppressor genes that escape X-chromosome inactivation [22]. In addition, experimental and molecular studies suggest possible sex-specific differences in responses to genotoxic stress and DNA damage repair, with women often showing higher expression of DNA repair genes and lower accumulation of somatic mutations during aging [23]. While these findings provide biologically plausible hypotheses for sex-related differences in cancer risk, direct evidence linking these mechanisms to observed population-level disparities in cancer incidence and mortality remains limited.

Sex hormones have also been implicated in cancer development and progression, although their effects vary substantially according to tumor type and biological context [24]. For example, estrogens have been suggested to promote the development of papilliferous cancer of the thyroid [25], whereas some epidemiological and experimental evidence has indicated a potential protective association with colorectal cancer [26]. In non-small cell lung cancer (NSCLC), experimental studies have shown that estradiol may activate the epidermal growth factor receptor (EGFR) pathway and upregulate CXCR4 expression, promoting cellular proliferation, angiogenesis, migration, and metastatic potential [27]. Similarly, sex hormones, including estrogens and androgens, have been proposed as modulators of susceptibility and tumor behavior in laryngeal cancer, although the clinical relevance of these mechanisms remains under investigation [28]. Emerging evidence further suggests that biological differences in hormonal regulation may influence susceptibility to tobacco- and alcohol-related carcinogenesis in women; however, the extent to which these mechanisms contribute to epidemiological sex disparities has not yet been fully established.

Beyond incidence and tumor biology, sex-related differences have also been observed in the tumor immune microenvironment. Women generally exhibit stronger innate and adaptive immune responses, a phenomenon that may be influenced by both hormonal factors and the enrichment of immune-related genes on the X chromosome. Incomplete X-chromosome inactivation has been proposed as one mechanism enhancing anti-tumor immunity in women, while loss or reduced expression of Y-linked genes has been associated with increased cancer susceptibility in men. Additional sex-biased differences have been reported in epigenetic regulation, cellular senescence, microbiota composition, metabolism, and DNA damage responses [29]. Although these observations support the existence of biologically meaningful sex differences, further research is needed to clarify their causal contribution to differences in cancer incidence, progression, treatment response, and mortality at the population level.

## 4. Differences in Therapies

Despite substantial advances in cancer therapies, optimizing treatment requires individualized dosing strategies that account for both biological sex and gender-related determinants, with the goal of maximizing efficacy while minimizing toxicity. Evidence from multiple international studies indicates that women are 1.5–2 times more likely than men to experience adverse drug reactions (ADRs) across all drug classes and are also more frequently hospitalized as a result. Current evidence suggests that these differences arise from the interplay of biological sex-related mechanisms affecting drug disposition and response, together with gender-related factors influencing treatment patterns, medication use, and adverse event reporting.

A variety of sex-related (biological) and gender-related (psychosocial and societal) variables seem to contribute to the disproportionately higher ADR susceptibility among women than men. These include sex-specific endogenous hormone exposure, sex-specific variations in exogenous sex hormone supplementation (e.g., oral contraceptives and menopausal hormone replacement therapies), sex-specific variations in gut microbiota composition, sex-specific variations in pharmacokinetics and pharmacodynamics, higher rates of polypharmacy in women, and gender differences in the reporting of ADRs, with women reporting more frequently.

Women exhibit significantly higher blood drug concentrations and longer drug clearance durations than men, which may be partly explained by women’s roughly 10% higher body fat, increased plasma volume, and organ perfusion. Because of the binding of drugs to erythrocytes, women’s lower hematocrit levels may possibly be a factor in this excessive drug toxicity. There may also be a role for sex differences in the expression levels of drug-metabolizing enzymes brought on by genetic polymorphisms (such as cytochrome P450 isoforms). Although information on the differential expression of distinct CYP450 isoforms either yields contradictory findings or does not show sex-based moderation, it has been reported that women exhibit 25% higher activity of the isoform CYP3A, which is responsible for the metabolism of around 50% of drugs. On the other hand, males have higher expression levels of the drug efflux pump P-gp, which is encoded by the MDR1 gene. This could help explain why men have a lower toxicity rate. In fact, by inhibiting P-gp activity in the rat small intestine, sex steroids were shown to control P-gp expression and improve drug absorption. There are significant sex differences in renal function (which is taken into account in renal function calculators), with men having an average of 20% greater renal function than women.

Several pharmacokinetic analyses have found that women have a lower elimination capacity for various anticancer drugs, including cytotoxic agents (i.e., paclitaxel, 5-fluorouracil [5-FU], and doxorubicin), tyrosine kinase inhibitors (i.e., imatinib and sunitinib), and monoclonal antibodies (i.e., bevacizumab and rituximab), which is associated with higher plasma levels. Male gender and body surface area are positively correlated with maximal elimination capacity of paclitaxel (VMEL); patient age and total bilirubin are negatively correlated with VMEL. Typically, male patients have a 20% higher VMEL; a 0.2 m^2^ increase in body surface area leads to a 9% increase in VMEL; a 10-year increase in patient age leads to a 5% decrease in VMEL; and a 10-μmol increase in total bilirubin leads to a 14% decrease in VMEL. Third-space effusions are not correlated with paclitaxel pharmacokinetics. Patient gender is shown to significantly and independently affect paclitaxel distribution and elimination. Body surface area, total bilirubin, and patient age were confirmed to affect paclitaxel elimination. About the relationship between 5-FU pharmacokinetics and toxicity, females have a slightly but significantly lower plasma clearance (CL) and a longer plasma half-life. Accordingly, the area under the plasma concentration (AUC) is higher in females than in males. Although these findings are in line with previous reports, conflicting data are also available in the literature. Indeed, one study could not demonstrate any sex-related differences in 5-FU kinetics, whereas another found that dihydropyrimidine dehydrogenase liver activity responsible for 5-FU catabolism was higher in females than in males, and our group reported that 5-FU CL, normalized by lean body mass instead of body weight (BW), was higher in females than in males. These apparent discrepancies may be reconciled by considering that 5-FU metabolism takes place not only in the liver but also in most metabolically active tissues and that lean body mass is less abundant in women than in men. Thus, when the dose is adjusted by body surface area (BSA) or BW, women receive a slightly higher dose per lean mass unit than men. Despite these well-documented sex-related differences, most analyses of anticancer drug elimination and distribution do not even include sex as a covariate. In a literature survey of 256 population studies on anticancer agents, only 80 reported that sex was included as a covariate in the analytic models [30].

The higher toxicity observed in women is likely to reflect both biological sex-related mechanisms and gender-related determinants.

A recent analysis of over 23,000 patients (38% women) enrolled in Phase II and III clinical trials demonstrated that female sex is associated with an increased risk of toxicity from anticancer therapies. Women had a 34% higher likelihood of experiencing severe adverse events compared with men (odds ratio [95% CI] 1.34 [1.27–1.42], *p* < 0.001). This elevated risk in women was consistent across treatment modalities (chemotherapy, targeted therapy, and immunotherapy), types of adverse events (symptomatic and hematologic), and treatment settings (advanced and adjuvant).

For instance, given average body type differences, women may receive a greater relative dose, although, importantly, we included covariate adjustment for obesity status to account for body type. It has been suggested that medication adherence for oral therapies may differ by sex, although this may not apply as much in the trial setting. Finally, biases may exist in the reporting or interpretation of adverse events (AEs), or men and women may differentially report AEs because of potential sex-related differences in symptom perception. Pharmacokinetics and pharmacodynamics could play a role. For the systemic therapies examined in this study, the literature is mixed. For instance, studies have found that women have a lower capacity to clear fluorouracil than men. By contrast, no differences between men and women in drug clearance were found in a study of patients receiving imatinib. Evidence is conflicting regarding the differential clearance rates of doxorubicin by sex. Pharmacogenetics (i.e., how drug metabolism and elimination of genetic polymorphisms modulate drug responses) may also vary by sex. For instance, survival in female patients with metastatic colon cancer treated with fluorouracil has been found to differ according to methylenetetrahydrofolate reductase gene polymorphism, but not in male patients. The protein ABCG2/BCRP/MXR/ABCP, an ATP-binding cassette transporter, influences the absorption, distribution, and excretion of drugs and may be regulated by sex hormones, with potential differential effects on drug toxicity for agents that interact with high affinity with ABCG2. The gut microbiome may also be implicated given its role in regulating metabolic and immune inflammatory pathways. It must take into account that the available evidence has limitations. First, clinical trial patients tend to be younger and healthier than non-trial patients on average, and therefore, toxicities may be greater in non-trial patients. Also, because only the worst toxicity grade in each category was recorded, the data do not allow for observation of toxicity patterns over time. Furthermore, toxicity must be considered in the context of survival; indeed, increased toxicity for women compared with men has been shown to occur in conjunction with improved survival. However, the causal relationship between AEs and survival may be challenging to interpret since improved survival may allow more time/exposure to develop AEs, or alternatively, increased AEs may represent, on average, increased delivery/efficacy of the anticancer agents, which could result in improved survival. In addition, the reporting of AE data may be subject to misclassification, especially when CTCAE criteria are unable to depict subtle symptoms. We also have to consider that some patients might have had existing sarcopenia, which might influence toxicity patterns and could not be fully accounted for by adjusting for obesity status. Finally, although pooling across clinical trial databases is necessary to enhance statistical power to identify trends in AEs by sex, it may also mask potentially meaningful associations.

In conclusion, underlying mechanisms may result in generalized worse toxicity outcomes for women, with or without corresponding survival improvements or detriments. Therefore, more awareness of symptom differences or reporting differences in women versus men is needed. A better understanding of the nature of the underlying mechanisms could potentially lead to interventions or delivery modifications to reduce toxicity, especially in women [31]. Importantly, the relationships between sex- and gender-related mechanisms and observed clinical differences remain largely associative, and causal pathways are not fully established.

Our recent review aimed to explore gender differences regarding treatments, especially in outcomes and toxicities related to therapies, and shows that women experience a lower survival benefit than men when treated with immunotherapy as monotherapy and a greater survival benefit when treated with a combination of immunotherapy and chemotherapy.

The evidence discussed below primarily concerns biological sex-related differences in antitumor immunity that may influence the efficacy of immunotherapy.

Evidence from the literature seems to show that therapy with anti-checkpoint T-lymphocyte-associated protein 4 (anti-CTLA-4) or anti-programmed cell death protein 1 (anti-PD-1) agents is more effective for men as compared with women in advanced cancers such as melanoma and non-small cell lung cancer. However, because the sex dimorphism of the immune system is complex, involving multiple elements of immune responses, it is possible that women could derive a larger benefit than men from strategies other than therapy with immune checkpoint inhibitors (ICIs) alone. Women with advanced lung cancer derive a statistically significantly larger benefit from the addition of chemotherapy to anti-PD-1/PD-L1 as compared with men. Sex-related differences in anticancer immune response have been described in the amount and composition of intratumoral immune infiltrates as well as in tumor expression levels of PD-L1 across a large spectrum of tumors, including NSCLC. An intratumoral immune infiltrate enriched with partially exhausted cytotoxic T lymphocytes (i.e., tumor-infiltrating CD8+ T cells expressing high levels of CTLA-4 and PD-1) strongly correlates with a higher response to anti-PD-1 monotherapy. It has been shown that intratumoral infiltrates of male patients with melanoma had a statistically significantly larger proportion of these cells compared with those of women. PD-L1 expression levels have been found to be statistically significantly higher in a number of solid tumors of female patients compared with those of men, including NSCLC. Given the complexity of the sex dimorphism of immune system function and responses, it would be possible that women derive a larger benefit than men from different immunotherapeutic strategies. Such heterogeneity of response is due to the ability of chemotherapy to increase the mutational burden and neoantigenic load of female lung cancer tumors that are statistically significantly lower than those of male tumors, with this also being a potential biological rationale that may help explain the lower efficacy of anti-PD-1 alone in women. Furthermore, the different efficacy of chemotherapy in modulating the anticancer immune responses of men and women could be speculated upon given the sex-related differences in the amount and composition of intratumoral immune infiltrates reported. As compared with men, the tumor microenvironment (TME) of women was significantly enriched for a number of innate and adaptive immune cell types, including specific T-cell subpopulations. NSCLCs of men and women exploited different mechanisms of immune evasion. The TME of females was characterized by significantly greater T-cell dysfunction status, higher expression of inhibitory immune checkpoint molecules, and higher abundance of immune-suppressive cells, including cancer-associated fibroblasts, myeloid-derived suppressor cells (MDSCs), and regulatory T cells. In contrast, the TME of males was significantly enriched for a T-cell-excluded phenotype. They reported data supporting impaired neoantigen presentation to the immune system in tumors of men as a molecular mechanism explaining the findings observed [32].

While these mechanisms provide biologically plausible explanations, they should be interpreted as hypotheses rather than established causal determinants of clinical outcomes. Overall, most of the evidence linking biological sex and gender-related determinants to clinical outcomes is observational, and mechanistic interpretations should be considered exploratory.

## 5. Cancer Care for the LGBTQI+ Community

While much of the evidence discussed so far has focused on biological sex differences influencing cancer susceptibility, tumor biology, treatment response, and treatment-related toxicity, the concept of gender oncology extends beyond biological determinants alone. It also encompasses the impact of gender identity, gender expression, and sociocultural factors on healthcare access, cancer prevention, treatment experiences, and clinical outcomes. Within this broader framework, understanding the specific needs and challenges of transgender, gender-diverse, and other LGBTQI+ populations is essential for achieving equitable and truly personalized cancer care.

Recent estimates suggest that between 0.3% and 0.5% of adults, and 1.2% to 2.7% of children and adolescents, identify as transgender, noting that these figures can vary by region and mainly derive from studies conducted in Anglo-Saxon and Northern European contexts [33].

Transgender individuals are defined as those whose gender identity differs from the societal expectations associated with the sex assigned to them at birth, while the term ‘gender diverse’ serves as a broad label for people whose gender identity or expression does not conform to established gender norms.

Cancer care is not exempt from the broader disparities in healthcare access and outcomes experienced by LGBTQI+ populations. Historically, these individuals have faced significant barriers, including discrimination, limited access to culturally competent care, and reduced availability of healthcare services. Social stigma and marginalization have further contributed to delayed diagnoses, lower participation in screening programs, and suboptimal treatment pathways.

Evidence highlights substantial gaps in cancer prevention practices. According to a recent report by the European Union Agency for Fundamental Rights, screening uptake among LGBTQI+ individuals remains lower than in the general population. In 2023, only 10% of LGBTQI+ individuals reported having undergone a mammogram in the previous year, compared with 36% of the general population. Similarly, 27% reported receiving a cervical smear test within the past 12 months, versus 36% in the general population. These disparities underscore the urgent need for more inclusive and accessible cancer prevention and care strategies [34]. The last available data about cancer screening adherence in Italy show that during the period 2018–2021, 79% of individuals assigned female at birth (AFAB) between 25 and 64 years had undergone a Pap test in the prior 5 years, 73% of AFAB between 50 and 69 years had received a mammography in the prior 2 years, and 47% of individuals between 50 and 69 years had undergone a procedure for colorectal cancer screening in the prior 5 years [35]. These data appear even more serious if we consider that higher rates of alcohol and tobacco use, as well as human papillomavirus (HPV) and human immunodeficiency virus (HIV) infection, are found in the LGBTQIA+ community, and as a result, a higher incidence of HIV- and HPV-associated cancers characterizes transgender and gender-diverse people compared to the cisgender population [36]. Furthermore, 2% of intersex people reported receiving a cancer diagnosis in the previous 12 months, which is greater than the 0.6% rate for the overall population [34].

Beyond disparities in cancer prevention and screening uptake, emerging evidence suggests that sexual and gender minority populations may also experience differences in cancer-related outcomes across the care continuum. Several studies, primarily from North America, have reported higher incidence rates of certain malignancies among transgender and gender-diverse individuals, particularly cancers associated with HIV and HPV infection, including anal, cervical, and oropharyngeal cancers. Delayed participation in screening programs and reduced access to culturally competent healthcare may contribute to later-stage diagnoses and more complex treatment pathways [37]. Evidence also indicates that LGBTQI+ patients frequently encounter barriers during cancer treatment, including misgendering, inadequate communication, a lack of provider knowledge regarding gender-affirming care, and concerns about discrimination within healthcare settings. These factors may negatively affect treatment adherence, patient satisfaction, psychological well-being, and overall quality of life. Cancer survivorship studies have similarly reported higher levels of distress, social isolation, and unmet supportive care needs among sexual and gender minority individuals compared with heterosexual and cisgender populations [38]. However, despite increasing recognition of these disparities, robust data on cancer-specific outcomes remain limited. Most available evidence originates from North American cohorts, while European data—and particularly data from Italy—are scarce. Furthermore, sexual orientation and gender identity variables are not routinely collected in cancer registries and clinical datasets, making it difficult to accurately assess differences in cancer incidence, stage at diagnosis, treatment delivery, recurrence, survival, and long-term outcomes [39]. Addressing these knowledge gaps through prospective and population-based research is essential to better understand the oncology-specific needs of LGBTQI+ populations and to develop evidence-based interventions aimed at reducing disparities.

Despite growing awareness of these disparities, research concerning cancer care and cancer-related outcomes among LGBTQI+ populations in Europe remains significantly limited, with the majority of information coming from North America and frequently failing to address the specific psychosocial effects or reactions to diagnosis faced by this group. There is a necessity for inclusive and intersectional research approaches that accurately represent the various identities within the LGBTQI+ population. There is a distinct demand for enhanced training for healthcare providers to foster cultural competence and minimize discrimination, as well as the development of targeted resources and campaigns to increase awareness of cancer risks that are particular to LGBTQI+ individuals. Furthermore, it is vital to develop inclusive cancer policies that cater specifically to the distinct needs of the LGBTQI+ community. Such policies must focus on removing the pathological labels from LGBTQI+ identities, tailoring medical treatment plans to individual patients, and making sure that screening initiatives are accessible to everyone. Applying these suggestions is essential for attaining fair cancer care and enhancing health outcomes for the LGBTQI+ population.

The American Society of Clinical Oncology (ASCO) has recognized sexual and gender minority (SGM) populations as a diverse group at increased risk of receiving inequitable care and experiencing discrimination across the cancer care continuum. Its position statement, “*Strategies for Reducing Cancer Health Disparities Among Sexual and Gender Minority Populations*,” calls for targeted outreach and educational support for SGM patients, enhanced cultural competency training for healthcare providers, the integration of sexual orientation and gender identity variables into quality-of-care metrics, and expanded data collection to better inform future interventions.

In Italy, the Italian Association of Medical Oncology (AIOM) has similarly acknowledged the importance of this issue. The “Assisi Recommendations” and the document “*Gender Oncology: Recommendations and Consensus of the Italian Association of Medical Oncology (AIOM)*” reflect the outcomes of the AIOM Oncology Ethics Day, which focused on gender differences in oncology and the care of transgender and gender-diverse individuals. Promoting research in this area, through the generation of robust evidence and the collection of prospective data, has been identified as a key priority to reduce existing disparities. By addressing the specific challenges faced by transgender and gender-diverse patients and advocating for dedicated clinical and policy guidance, AIOM underscores its commitment to ensuring equitable cancer care for all, regardless of gender identity or social context, in line with a broader “health for all” vision.

More broadly, medical oncology is increasingly moving toward a truly personalized model of care, one that considers the full spectrum of individual patient characteristics, including biological, social, and behavioral factors, among which sexual orientation and gender identity may also influence disease trajectory and care outcomes.

## 6. Conclusions

Sex (e.g., chromosomes, hormones, and immune function) and gender (e.g., social roles, behaviors, and access to care) both play a critical role in shaping cancer incidence, progression, and survival. Across most malignancies affecting both sexes, men show higher incidence and mortality rates, and this pattern is consistently observed worldwide and reflected in national data, despite variations between countries.

Sex- and gender-based differences are also evident in treatment outcomes and toxicity profiles. Women experience a lower survival benefit when treated with immunotherapy as monotherapy, but a greater survival benefit is observed in women treated with a combination of immunotherapy and chemotherapy. At the same time, women experience a higher burden of treatment-related adverse events, particularly with immunotherapy and fluoropyrimidine-based regimens such as 5-fluorouracil. These differences are likely driven by variations in hormonal pathways, immune responses, drug metabolism, and genetic and epigenetic factors. In parallel, gender-related determinants, including smoking and alcohol use, occupational exposures, health-seeking behaviors, and participation in screening programs, further contribute to disparities in cancer detection and outcomes.

Gender pharmacology is a complex field of study, requiring consideration not only of biological and physiological differences but also of psychosocial, cultural, and behavioral factors that shape individual health trajectories. Despite this, few medicinal products include sex-specific information in their prescribing data, largely due to the historical underrepresentation of women in clinical trials. Advancing precision oncology will require a stronger commitment to inclusive research, including increased enrollment of women, systematic sex- and gender-disaggregated analyses, and the development of tailored therapeutic strategies, such as optimized dosing and scheduling.

Beyond the binary framework of male and female, sexual and gender minority (LGBTQIA+) populations continue to face significant inequities in access to healthcare and cancer outcomes. Addressing these disparities is essential not only from an ethical perspective but also to improve overall cancer control. Greater awareness and targeted action within the oncology community—particularly in Europe—are needed to ensure equitable, inclusive, and high-quality cancer care for all populations, including other marginalized groups.

## Data Availability

No new data were created or analyzed in this study. Data sharing is not applicable to this article.

## Abbreviations

NSCLC = non-small cell lung cancer, EGFR = epidermal growth factor receptor, ADR = adverse drug reaction, VMEL = maximal elimination capacity of paclitaxel, 5-FU = 5-fluorouracil, CL = plasma clearance, AUC = area under the plasma concentration, BW = body weight, BSA = body surface area, AE = adverse event, anti-CTLA-4 = anti-checkpoint T-lymphocyte-associated protein 4, anti-PD-1 = anti-programmed cell death protein 1, ICI = immune checkpoint inhibitor, TME = tumor microenvironment, AFAB = assigned female at birth, SGM = sexual and gender minority, HPV = human papillomavirus, HIV = human immunodeficiency virus, ASCO = American Association of Clinical Oncology, SGM = sexual and gender minority, AIOM = Italian Association of Medical Oncology.
